# Chess AI: Competing Paradigms for Machine Intelligence

**DOI:** 10.3390/e24040550

**Published:** 2022-04-14

**Authors:** Shiva Maharaj, Nick Polson, Alex Turk

**Affiliations:** 1 ChessEd, 729 Colby Ct, Gurnee, IL 60031, USA; problemsolver2020@gmail.com; 2 Booth School of Business, University of Chicago, Chicago, IL 60637, USA; 3 Phillips Academy, Andover, MA 01810, USA; alexwturk@gmail.com

**Keywords:** AI, AGI, AlphaZero, LCZero, Bayesian, chess, chess studies, neural networks, Plaskett’s study, reinforcement learning

## Abstract

Endgame studies have long served as a tool for testing human creativity and intelligence. We find that they can serve as a tool for testing machine ability as well. Two of the leading chess engines, Stockfish and Leela Chess Zero (LCZero), employ significantly different methods during play. We use Plaskett’s Puzzle, a famous endgame study from the late 1970s, to compare the two engines. Our experiments show that Stockfish outperforms LCZero on the puzzle. We examine the algorithmic differences between the engines and use our observations as a basis for carefully interpreting the test results. Drawing inspiration from how humans solve chess problems, we ask whether machines can possess a form of imagination. On the theoretical side, we describe how Bellman’s equation may be applied to optimize the probability of winning. To conclude, we discuss the implications of our work on artificial intelligence (AI) and artificial general intelligence (AGI), suggesting possible avenues for future research.

## 1. Introduction

*Chess is not a game. Chess is a well-defined form of computation. You may not be able to work out the answers, but in theory, there must be a solution, a right procedure in any position—John von Neumann*

There are various schools of thought in chess composition, each of which placing different emphasis on the complexity of problems. Chess studies originally became popular in the 19th century. They are a notoriously difficult kind of puzzle, involving detailed calculations and tactical motifs. As such, they provide a good benchmark for AI studies.

We choose to analyse how chess engines respond to Plaskett’s Puzzle, one of the most well-known endgame studies in history. The endgame is the final phase of a chess game. There are many features of Plaskett’s Puzzle that make it particularly hard—not just the depth required (15 moves) but also the use of underpromotion (a particularly counterintuitive kind of move in chess) and subtle tactical strategies. Though the puzzle was initially created by a Dutch composer, it achieved notoriety in 1987 when English grandmaster Jim Plaskett posed the problem at a chess tournament, stumping all players except Mikhail Tal.

The advent of powerful computer chess engines enables us to revisit chess studies where the highest level of tactics and accuracy are required. We evaluate the performance of Stockfish 14 [1] and Leela Chess Zero (LCZero) [2] on Plaskett’s Puzzle. We discuss the implications of their performance for both end users and the artificial intelligence community as a whole. We find that Stockfish solves the puzzle with much greater efficiency than LCZero. Our work suggests that building a chess engine with a broad and efficient search may still be the most robust approach.

Chess engine research has implications for problems requiring large-scale tree search. LCZero and its predecessor, AlphaZero [3], use neural networks to guide a Monte Carlo tree search. Researchers have since used this idea to construct procedures for chemical synthesis [4] and optimize the dynamics of quantum systems [5]. Stockfish heavily relies on alpha-beta search [6], which has been used to verify neural networks’ robustness to adversarial attacks [7].

Artificial general intelligence (AGI) is the ability of an algorithm to achieve human intelligence in a multitude of tasks. Ref. [8] famously claimed that the rules of chess could be determined from empirical observation, a central tenet of artificial general intelligence. Historical records provide insight into human performance on Plaskett’s Puzzle. By comparing this with machine performance, we can better assess the state of current progress on the long road to building AGI. We further discuss which algorithms possess greater potential in the field of AGI by reviewing their ability to generalize to different domains.

On the theoretical side, we describe a central tool for solving dynamic stochastic programming problems: the Bellman equation. This framework, together with the use of deep neural networks to model the value and policy functions, provides a solution for maximising the probability of winning the chess game. In doing so, the algorithm offers an optimal path of play. Given the enormous number of possible paths of play (the Shannon number), the use of search methods and the depth of the algorithm become important computational aspects. It is here that high-level chess studies provide an important mechanism for testing the ability of a given AI algorithm.

The rest of the paper is outlined as follows. The next subsection provides historical perspective on Plaskett’s Puzzle. Section 2 provides background material on current AI algorithms used in chess, namely, Stockfish 14 and LCZero. Section 3 describes our software configuration and experimental approach. Section 4 provides our detailed analysis of Plaskett’s Puzzle using these chess engines. Finally, Section 5 concludes with directions for future research. In particular, we discuss the implications of our work for human–AI interaction in chess [9] and AI development.

### Plaskett’s Study

The story of Plaskett’s Puzzle dates to a Brussels tournament in 1987. The study was originally composed by Gijs van Breukelen in 1970. In 1987, it famously stumped multiple super grandmasters—players at the highest level in chess—when presented by James Plaskett in a tournament press room. It was finally solved that day by legendary attacking player Mikhail Tal, who figured it out during a break at the park [10].

**Figure 1:**
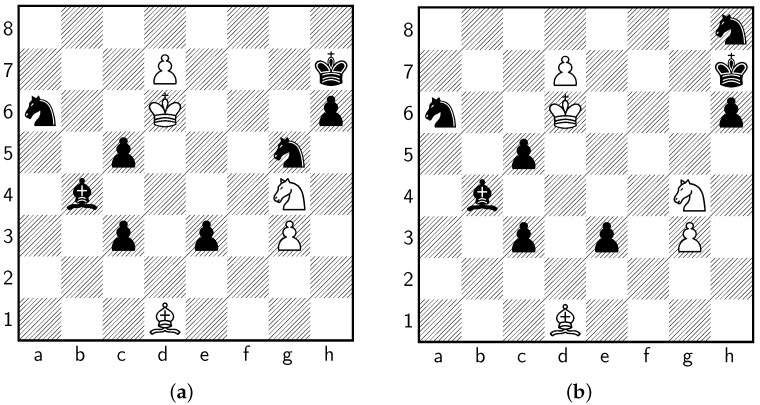
Versions of Plaskett’s Puzzle. (**a**) The original Plaskett’s Puzzle. (**b**) The corrected Plaskett’s Puzzle.

The highly inventive puzzle involves multiple underpromotions and was originally designed to be a checkmate in 14. There is a mistake in the original puzzle whereby Black can escape checkmate, though White is still winning in the final position. This mistake can be corrected by moving the Black knight on g5 to h8. Harold van der Heijden’s famous *Endgame Study Database* (which contains over 58,000 studies) proposes another corrected version where he moves the Black knight from g5 to e5. Figure 1a shows the original Plaskett’s Puzzle, while Figure 1b shows the corrected Plaskett’s Puzzle.

## 2. Background: Chess AI

Refs. [11,12,13] pioneered the development of AI algorithms for chess. The Shannon number, $10^{120}$, provides a lower bound on the total number of possible games, making chess a daunting computational challenge. A major advance over pure look-ahead search methods was the use of deep neural networks to approximate the value and policy functions. Then, the self-play of the algorithms allowed for quick iterative solution paths. See work by [14] for further discussion.

The dynamic programming method breaks the decision problem into smaller subproblems. Bellman’s principle of optimality describes how to do this:

*Bellman Principle of Optimality: An optimal policy has the property that whatever the initial state and initial decision are, the remaining decisions must constitute an optimal policy with regard to the state resulting from the first decision.*

[15]

Backwards induction identifies what action would be most optimal at the last node in the decision tree (i.e., checkmate). Using this information, one can then determine what to do at the second-to-last time of decision. This process continues backwards until one has determined the best action for every possible situation (i.e., solving the Bellman equation).

### 2.1. Q-Values

The optimal sequential decision problem is solved by calculating the values of the *Q*-matrix, denoted by Q(s,a) for state *s* and action *a*. One iterative process for finding these values is known as *Q*-learning [16], which can be converted into a simulation algorithm as shown by [17]. The *Q*-value matrix describes the value of performing action *a* (a chess move) in our current state *s* (the chess board position) and then acting optimally henceforth. The current optimal value and policy function are as follows:

(1) $$\begin{array}{cc} \; & V\left(s\right)=\max_{a}\;Q\left(s,a\right)=Q\left(s,a^{★}\left(s\right)\right) \end{array}$$

(2) $$\begin{array}{cc} & a^{★}\left(s\right)={\arg\max}_{a}\;Q\left(s,a\right). \end{array}$$

For example, chess engines such as LCZero simply take the probability of winning as the objective function. Hence, at each stage, V(s) measures the probability of winning. This is typically reported as a pawn advantage, since the pawn is the most basic unit of material in chess. Advantages based on the arrangement of the pieces are heuristically factored into this metric as well. The greater a side’s pawn advantage, the greater their chance of winning. Ref. [18] provides an equation for converting between pawn advantage and win probability.

The Bellman equation for *Q*-values (assuming instantaneous utility u(s,a) and a time-inhomogeneous *Q* matrix) is the constraint

(3) $$Q\left(s,a\right)=u\left(s,a\right)+\sum_{s^{★}\in S}P\left(s^{★}|s,a\right)\max_{a}Q\left(s^{★},a\right).$$

Here, $P(s^{★}|s,a)$ denotes the transition matrix of states and describes the probability of moving to a new state $s^{★}$ given the current state *s* and action *a*. The new state $s^{★}$ is the board position after the current player has played action *a* and the opponent has responded. Bellman’s optimality principle is therefore simply describing the constraint for optimal play as one in which the current value is a sum over all future paths of the probabilistically weighted optimal future values of the next state.

Taking the maximum value of Q(s,a) over the current action *a* yields

(4) $$V\left(s\right)=\max_{a}\left\{u\left(s,a\right)+\sum_{s^{★}\in S}P\left(s^{★}|s,a\right)V\left(s^{★}\right)\right\}\;\;\mathrm{where}\;\;V\left(s^{★}\right)=\max_{a}\;Q\left(s^{★},a\right).$$

Here, u(s,a) is an instantaneous utility obtained from action *a* in state *s*. For all nonterminal board positions, u(s,a) is zero. At the end of the game, u(s,a) is −1 after a loss, 0 after a draw, and 1 after a win.

### 2.2. Stockfish 14 Anatomy

#### 2.2.1. Search

Stockfish uses the alpha-beta pruning search algorithm [6]. Alpha-beta pruning improves minimax search [12,19] by avoiding variations that will never be reached in optimal play because either player will redirect the game.

Since it is often computationally infeasible to search until the end of the game, the search is terminated early when it reaches a certain depth. Search depth is measured by *ply*, where a ply is a turn taken by a player. A search depth of *D* indicates that the distance between the root node and the leaf nodes of the search tree is *D* plies. Stockfish incrementally increases the depth of its search tree in a process known as *iterative deepening* [20]. However, when a nominal search depth of *D* is reported by chess engines, it does not mean that the search has considered all possible variations of *D* moves. This is due to heuristics which cause the engine to search promising variations to a greater depth than nominal and less promising variations to a lesser depth than nominal.

The engine applies two main classes of heuristics to reduce the search space: forward pruning and reduction [21]. Forward pruning techniques remove game tree subgraphs that are unlikely to be contained in optimal play. For example, if the evaluation of a position is significantly worse than the value guaranteed by a player’s best alternative, the position’s children are pruned early. This is known as futility pruning [22,23]. It is possible that the engine mistakenly prunes a line of play. This will be corrected once the engine depth surpasses a technique-specific depth cap (see Stockfish source code at https://github.com/official-stockfish/Stockfish, accessed on 13 April 2022); after which, the technique is no longer applied.

Reduction techniques search certain game tree subgraphs to lower depths, rather than omitting them from the search altogether. A canonical example is late move reductions [24], which assume that the engine checks better moves earlier. Moves checked later are searched to lower depths than nominal.

Given infinite time, the engine will converge to the optimal line of play. Depth caps prevent lines from being overlooked via pruning, and reductions become inconsequential at infinite depth.

#### 2.2.2. Evaluation

Once the search algorithm reaches a leaf node, a heuristic evaluation function is applied to determine whether the ending position favours White or Black. In Stockfish versions 11 and under, this function is hard-coded based on chess concepts such as piece position, piece activity, and the game phase (opening/middle game or endgame) (see https://hxim.github.io/Stockfish-Evaluation-Guide/, accessed on 13 April 2022).

The efficiently updatable neural network (NNUE) evaluation function was originally invented by [25] for Shogi, a Japanese chess variant. Stockfish implemented it for their chess engine in version 12 [26]. The neural network is trained to predict the output of the classic Stockfish evaluation function at “moderate” search depths. Thus, it can be thought of as a search depth multiplier. Though NNUE is about twice as slow as Stockfish’s classic evaluation function, the engine makes up for this in terms of evaluation quality [27].

Its architecture comprises a shallow, four-layer neural network specifically optimized for speed on CPU machines. The input layer of binary features describes the position of the White and Black king relative to the White and Black pieces, respectively. For example, one feature might be described as

(5) $$\begin{array}{c} \theta_{0}=\left\{\begin{array}{cc} 1 & \mathrm{if}\;\mathrm{Black}\;♔g8\;\mathrm{and}\;♕d8\\ 0 & \mathrm{otherwise}. \end{array}\right. \end{array}$$

The dimensionality of the board feature vector is reduced as it passes through two hidden layers and one output layer, resulting in a final scalar value for the position’s evaluation.

### 2.3. AlphaZero Anatomy

#### 2.3.1. Search

AlphaZero uses the Monte Carlo tree search (MCTS) algorithm to identify the best lines via repeated sampling [3]. In MCTS, node evaluations at the end of prior simulations are used to direct future simulations toward the most promising variations. The search tree starts with one node (the current position). Each simulation traces a path through the tree according to node values, expanding the tree by a sampling an additional node with high prior probability once it reaches the end. After evaluating this new node with the evaluation function, a backward pass through the tree is performed to update the *Q*-values of nodes visited during the current simulation. After some number of simulations, the child node with the most samples is deemed the algorithm’s choice. During simulation, nodes are optimistically selected according to the polynomial upper confidence tree (PUCT) algorithm [14,28]:

(6) $$\begin{array}{c} \begin{array}{cc} \; & a_{t}=\underset{a}{\mathrm{error}}\left(Q\left(s_{t},a\right)+U\left(s_{t},a\right)\right)\\ \; & U\left(s,a\right)=C\left(s\right)P\left(s,a\right)\frac{\sqrt{\sum_{b}N\left(s,b\right)}}{1+N(s,a)}\\ \; & C\left(s\right)=\log\frac{1+N\left(s\right)+c_{base}}{c_{base}}+c_{init}. \end{array} \end{array}$$

Both the action value Q(s,a) and policy P(s,a) are determined by applying the evaluation function to states in the game tree. The Q(s,a) is the mean action-value of the node across all simulations, while P(s,a) is the prior probability of the node according to the policy network. N(s,a) is the number of times the action *a* has been taken from state *s*. The search algorithm initially focuses on nodes with high prior probability *P*. As N(s,a) increases, the algorithm increasingly relies on the sampled *Q*-value. C(s) controls the amount of exploration, which increases as the search progresses. Constants $c_{base}$ and $c_{init}$ thus alter the rate of exploration during the search.

#### 2.3.2. Evaluation

AlphaZero’s evaluation uses deep convolutional neural networks (CNNs) to estimate the policy vector $p_{t}=P\left(a|s_{t}\right)$ and value $v_{t}$ of nodes in the search tree [3]. Data are generated through millions of games of self-play, where AlphaZero plays both sides. Self-play removes reliance on human experts, and through many games, AlphaZero is able to correct its mistakes and discover novel game-playing strategies. Given the final outcome of a game *z* (−1 for loss, 0 for draw, or +1 for win) and the search probabilities $\pi_{t}$ (obtained from the final node visit counts), the networks are trained to minimize the loss of

(7) $${\left(z-v_{t}\right)}^{2}-\pi_{t}^{T}\log p_{t}+c{∥\theta ∥}^{2}$$

where θ represents the parameters and *c* controls the amount of regularization. While *v* aims to directly predict the game result, p is trained to “look ahead” in the search, causing it to learn helpful prior probabilities for the PUCT algorithm.

Early neural chess engines often required hand-crafted feature representations that went beyond the board position and basic board statistics. For example, the NeuroChess [29] input features included the number of weak pawns on the board and the relative position of the knight and queen. With the advent of deep learning, the process of performing feature selection by hand can be eliminated, as additional layers perform a sophisticated—and perhaps even more effective—form of feature selection [30]. The learner relies on the “unreasonable effectiveness of data” to learn useful features from unstructured input [31].

In particular, AlphaZero’s CNN inputs are binary 8×8 feature planes that encode the piece locations of both players for the eight past half-moves. In addition, the input includes constant feature planes which encode important counts in the game, such as the repetition and move count. Like the input, the output policy $p_{t}=P\left(a|s_{t}\right)$ is represented in the network by a stack of planes. Each of the planes in the 8×8×73 stack represents a movement-related modality, and each square represents the location from which to pick a piece up. For example, the first 8×8 plane might represent the probabilities assigned for moving one square north from each of the squares on the board. Illegal moves are filtered out before the probability distribution over all 4672 possible moves is computed.

The body of the network consists of 19 residual blocks [32] made up of rectified convolutional layers and skip connections. The network body leads into two “heads”: a value head, which produces the scalar evaluation, and a policy head, which produces a stack of planes as detailed above. Ref. [3] provides further details.

### 2.4. AlphaZero’s Successor: LCZero

DeepMind did not open-source AlphaZero. The LCZero project was thus born as an attempt to reproduce the work via crowd computing. Though LCZero predominantly uses the same search and evaluation techniques as AlphaZero, the team has made a few improvements. For example, the network architecture adds squeeze-and-excitation layers [33] to the residual blocks, and the engine supports endgame tablebases. The developers also added a moves-left network, which predicts the remaining game length in plies and helps LCZero navigate endgame positions more effectively. LCZero has far surpassed the original strength of AlphaZero due to its additional training and improvements.

## 3. Materials and Methods

Both Stockfish and LCZero are freely available online. Stockfish is available at https://stockfishchess.org, accessed on 13 April 2022, and LCZero is available at https://lczero.org, accessed on 13 April 2022. In our tests, we used Stockfish version 14 and LCZero network number 609950. We used the default settings for both engines. For a given position, we report the engine’s top 5 moves along with their corresponding pawn advantages and win probabilities. Though neither Stockfish nor LCZero are stochastic during evaluation by design, multithreading introduces randomness into the evaluations of both engines. Thus, we average our results over five trials and report standard deviations. In addition, we empirically analyse variations generated by the Stockfish and LCZero search procedures.

## 4. Results

### 4.1. Stockfish 14 Performance

We gain insight into how Stockfish 14 understands Plaskett’s Puzzle by inspecting the action-value function *Q* for the top moves.

Table 1 shows that given the original puzzle (see Figure 1a), Stockfish prefers Black, reporting a pawn advantage of −3.62. It does not identify the winning move in its top five choices. However, the engine’s evaluation of the position reverses once it analyses at a greater depth, as illustrated in Table 2.

**Figure 2:**
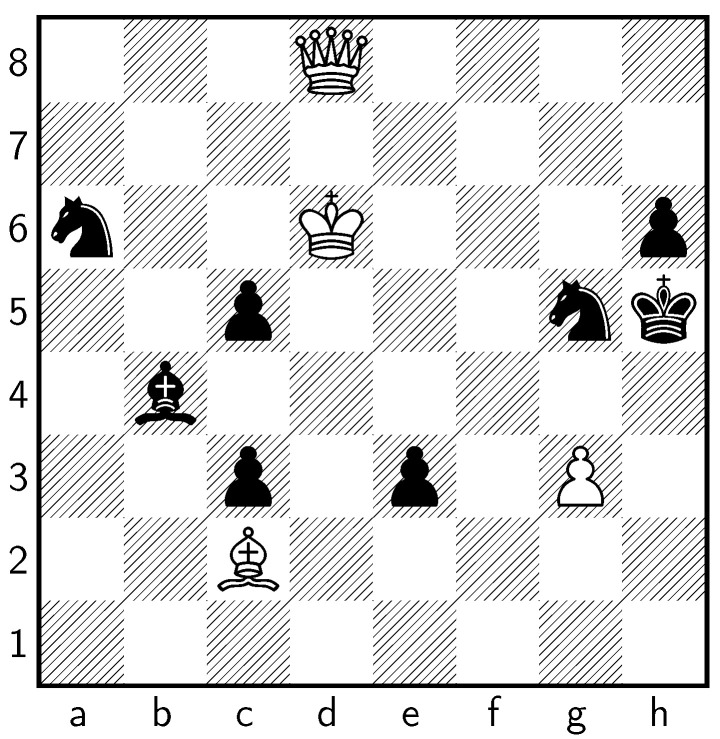
The position after the first four moves of Van Breukelen’s intended continuation.

Van Breukelen originally designed the solution of the puzzle to begin with **1 ♘f6+ ♔g7 2 ♘h5+ ♔g6 3 ♗c2+! ♔×h5 4 d8♕**. The resulting position is depicted in Figure 2.

The principal variation identified by Stockfish exploits a flaw in the original study. The engine correctly calculates that Black can avoid walking into Van Breuklen’s forced checkmate with **4...♔g4! 5 ♕f6 ♔×g3 6 ♕f1 c4+ 7 ♔d5**. If **♘f7+** is played instead, it leads to a forced checkmate via **4...♘f7+ 5 ♔e6 ♘×d8+ 6 ♔f5 e2 7 ♗e4 e1♘! 8 ♗d5! c2 9 ♗c4 c1♘! 10 ♗b5 ♘c7 11 ♗a4! ♘e2 12 ♗d1 ♘f3 13 ♗×e2 ♘ce6 14 ♗×f3⌗**.

Testing Stockfish against the corrected puzzle (depicted in Figure 1b), we obtain similar results, which are reported in Table 3. Though the forced checkmate is 29 half-moves deep, the chess engine reaches around a nominal depth of 40 before it detects the move.

For both the original and corrected puzzles, Stockfish’s delay in finding the move is due to the heuristics that the engine uses for focusing on more promising branches. For example, it is likely that the knight sacrifice with **3 ♗c2+** is sorted near the end of the move list, since alternative moves leave White with more material. Therefore, late move reductions will cause the engine to search variations following **3 ♗c2+** to less depth than nominal.

### 4.2. LCZero Performance

We test LCZero on the corrected puzzle and find that, even with an extensive amount of computational resources, it does not find the forced checkmate. The engine’s evaluations are provided in Table 4.

**Figure 3:**
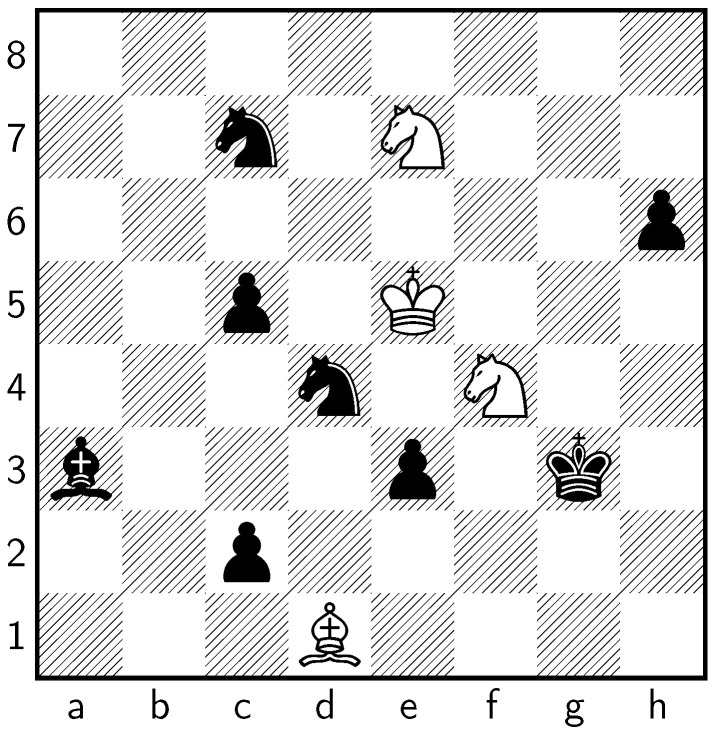
The final position after LCZero’s continuation following **1 ♘f6+**.

The engine reports that the best move for Black is **1 d8♖** with a pawn advantage of −4.67. It further assigns the winning move, **1 ♘f6+**, a pawn advantage of −5.61 and calculates the full continuation as **1 ♘f6+ ♔g7 2 ♘h5+ ♔g6 3 d8♘ ♔f5 4 ♘f4 ♔e4 5 ♘c6 ♘f7+ 6 ♔e6 ♘g5+ 7 ♔f6 ♘c7 8 ♘e7 ♘ge6 9 ♗c2+ ♔f3 10 ♗d1+ ♔×g3 11 ♘d3 ♘d4 12 ♔e5 ♗a3 13 ♘f4 c2**. The resulting position for this continuation is shown in Figure 3.

The ending position is clearly winning for Black.

It is possible to understand Leela’s selective search strategy by examining the distribution of positions searched. A surprising 92.4% of the 60 million searched positions follow from **1 ♘×e3**, even though its win probability of 3.01% is lower than the top 5 moves. On average, the engine spends 0.36% of the time, or about 216,000 nodes (SD = 450), searching positions following **1 ♘f6+**, seeing it as less promising. This is partially due to the prior probabilities determined by the policy head. The policy indicates that there is a 15.75% probability **1 ♘×e3** is the optimal move, compared to a 7.40% probability for **1 ♘f6+**. This intuition seems reasonable, since Stockfish evaluates **1 ♘×e3** as the second-best move in the position, as shown in Table 3. Furthermore, once LCZero determines that all moves seem losing, it tries to focus on the one with the most promise to extend the game, which the moves-left head identifies as **1 ♘×e3**. It stands to reason that a node with significantly more moves remaining would also require a deeper search (there is greater potential the engine will stumble upon a line that will reverse its evaluation deep in the tree). However, the skewed search is also due to subtleties in the puzzle. The engine must see the entire checkmate before it is able to realize the benefits, especially regarding the **3 ♗c2+** sacrifice made in a materially losing position. The engine thus chooses to prioritize searching other lines instead.

LCZero finds the forced checkmate after it is given the first move. This happens after searching about 5.5M nodes. In the same position, Stockfish searches around 500 M nodes before finding the checkmate. This demonstrates LCZero’s strength: even if Stockfish searches significantly faster than LCZero, LCZero can find the optimal line of play after searching orders of magnitude less nodes.

### 4.3. Fairness of Engine Comparison

Historically, engines have been compared by running them on the same hardware. However, the advent of GPU engines has meant that this is not always possible anymore. CPU engines run suboptimally on GPUs, and GPU engines run suboptimally on CPUs. The closest we can do is establish rough equivalences between GPU and CPU hardware.

The “Leela ratio” factor is based on the ratio of the GPU and CPU evaluation speeds reported in the AlphaZero paper [3] (see https://github.com/dkappe/leela-ratio, accessed on 13 April 2022). Its use assumes that the Stockfish vs. AlphaZero games ran on “equivalent” hardware. By comparing the GPU and CPU evaluation speeds on our hardware to the Leela Ratio, it is possible to determine whether we give an edge to either engine.

We report and recompute an updated Leela Ratio based on the average engine speeds (in nodes per second) from the Top Chess Engine Championship (TCEC) Cup 8 Final held in March 2021. Our comparison thus assumes that the TCEC chess engine tournament picks “fair” hardware when it pits CPU and GPU engines against one another. We recompute the Leela ratio factor *F* as

(8) $$F=\frac{\mathrm{Stockfish}\;\mathrm{nps}}{\mathrm{LCZero}\;\mathrm{nps}}=\frac{1.5\times 10^{8}}{1.4\times 10^{5}}\approx 1084.$$

This means that during TCEC, Stockfish is allowed to search, on average, *three orders of magnitude* more nodes than LCZero in “fair play”. Based on this ratio, we calculate the Leela ratio for our experiments as

(9) $$R=F\times \frac{\mathrm{LCZero}\;\mathrm{nodes}}{\mathrm{Stockfish}\;\mathrm{nodes}}=F\times \frac{6.0\times 10^{7}}{1.897\times 10^{9}}\approx 34.$$

This indicates that we gave LCZero 34 times more computational power than we gave Stockfish (compared to TCEC). Our experiments show that even with this advantage, LCZero does not find the solution to the corrected Plaskett’s Puzzle, while Stockfish does.

## 5. Discussion

Stockfish and LCZero represent two competing paradigms in the race to build the best chess engine. The magic of the Stockfish engine is programmed into its search, the magic of LCZero into its evaluation. When tasked with solving Plaskett’s Puzzle, Stockfish’s approach proved superior. The engine searched through nearly 1.9 billion different positions to identify the minimax solution. The algorithm’s sheer efficiency—due in part to domain-specific search optimizations—enabled it to find the surprising, unlikely solution. On the other hand, LCZero’s selective search was less efficient, primarily because it chose the wrong lines to search deeply. Even its more powerful, deep-learning-based evaluation function failed to recognize the positional potential of the knight sacrifice. This is particularly notable because LCZero’s evaluation function is often unafraid of giving away material for a positional advantage.

Since engines often cannot compute to the end of the game, there is uncertainty in the values they assign to moves. The goal of a forward search from some position *s* is to reduce the uncertainty in Q(s,a) for each action *a*. This uncertainty can be represented as a distribution over all possible *Q*-values an action could take. More uncertainty leads to a greater continuous entropy for the distribution over possible *Q*-values [34]. During search, the PUCT algorithm implicitly models and reduces the amount of uncertainty in the engine’s reported *Q*-values. Comparing search algorithms may thus be viewed as comparing different ways that engines resolve uncertainty in their evaluations.

After annotating 40 games between Stockfish and LCZero, FIDE Chess Master Bill Jordan concluded that “Stockfish represents calculation” and “Leela represents intuition” [35]. For experienced human players, decisions that might have priorly required “logical apparatus” become ingrained to the point of “automation” through repetition [36]. Like human players, LCZero’s intuition allows it to hone in on the most compelling lines. In the majority of positions, this intuition is strong enough to make up for fewer calculations. However, LCZero’s human-like approach can fail in edge cases; it is difficult to build a universally accurate pattern matcher. The safest approach to engine-building may still involve cold, hard calculation, even in seemingly unpromising variations.

That said, there is another aspect of LCZero’s algorithm that ought to be considered. The engine was built from “zero” knowledge of the game, other than the rules. AlphaZero’s successor, MuZero [37], removed the dependence on rules altogether [8]. It stands to reason that with work, LCZero could be trained with this method as well. Such an approach stands in sharp contrast to the large quantity of chess-specific search heuristics required by Stockfish. AlphaZero additionally achieves high levels of performance in Shogi and Go, which demonstrates that the LCZero algorithm is significantly more *generalizable* than Stockfish. In games where humans do not have the knowledge or time to provide domain-specific information, a strong engine can still be trained.

Stockfish and LCZero are examples of what philosopher John Searle calls weak AI [38]. Weak AI is a tool that performs a task that the mind can perform. This is in contrast to strong AI, which singularly possesses the capabilities of the human mind. Strong AI *understands* the world in the same way that humans do and is often known as artificial general intelligence, or AGI.

Since LCZero learns to play chess without additional human knowledge, its approach demonstrates more potential in the field of AGI. However, the engine’s inability to solve Plaskett’s Puzzle (given a reasonable amount of resources) means that even the strongest versions of weak AI have not been able to replicate expert human performance. Therefore, we propose two main courses of action for improving LCZero’s efficiency on the problem.

One potential solution is additional training. With more self-play, it is likely that LCZero’s performance on the puzzle would improve. It may further be possible to train the engine on forced checkmates, which would develop its pattern recognition abilities in mating positions. Such an approach seeks to refine the output of LCZero’s policy head through data augmentation [39], improving the engine’s intuition. Unlike training on expert games, this form of training is impervious to human bias, since the optimal line of play is already known.

Our second proposal involves algorithmic and architectural improvement. Improvements to the search, for example, have already begun with Ceres, which selects lines more intelligently [40]. Neural architecture improvements can be explored by experimenting with ideas that have seen widespread success in natural language processing, such as attention [41] and transformers [42,43]. Speed-ups might also be gained by simplifying the neural network architecture through different forms of model compression. Common methods include knowledge distillation [44,45], neuron pruning [46,47], and quantization [48]. With a faster evaluation function, LCZero can search through more nodes, increasing the likelihood that it will find the optimal line of play.

For chess-problem-specific algorithmic improvement, we draw inspiration from how humans solve chess problems. The human approach may not always be the most effective one [49], but it provides us with a starting point. We notice that humans solve chess problems not only with calculation but also with *imagination*. In the case of Plaskett’s Puzzle, it is possible that Tal saw the checkmate in his mind *first* and then found the variation that led him there. We ask whether machines can possess a similar imagination [50].

Since all puzzles have a guaranteed solution, it may be possible for a learner to explicitly predict likely checkmate positions and use these positions to condition the search. Such a process would augment the sequential search process with a deeper, more speculative lookahead. In statistical terms, this may be thought of as “extending the conversation”, since we are extending our assessment of win probability by conditioning on the event of reaching a certain checkmate position [51]. It is important to note that in order to verify a checkmate as a forced win, all relevant lines must still be examined. The opponent must truly have no alternative. Humans often optimistically bias their calculations based on their memory of similar positions. They may fall into the trap of “magical thinking”, which “involves our inclination to seek and interpret connections between the events around us together with our disinclination to revise belief after further observation” [52]. When implementing a computational form of imagination, it is important to ensure machines do not make the same mistake.

To make progress in AGI, however, systems must do more than improve on a particular task; they must begin to generalize better to new tasks. Ref. [3] took an important step in this direction by building a singular architecture that achieves state-of-the-art results in three different games. Such work begs the question of what a singular model can achieve. Thus, a compelling avenue of research resides in testing changes in performance when AlphaZero or LCZero learns to play multiple games at once. This can be achieved via multitask learning [53], which has seen success in both NLP [54] and computer vision [55].

For end users, our work implies that Stockfish may currently be the better tool for studying deep puzzles. To confirm this hypothesis, a chess studies dataset ought to be created and used as a benchmark for modern chess engines. Furthermore, different parameter combinations for chess engines ought to be tested to determine whether performance improvements can be gained. For the AI community, our work suggests that even extremely advanced incarnations of weak AI do not outperform humans in all cases. More research will be critical to improving these systems, particularly as we begin to approach the larger goal of building strong AI.

## Figures and Tables

**Table 1:** Original puzzle: Stockfish 14 evaluations at depth 30 for the top 5 moves (MultiPV = 5) and corresponding approximate win probabilities (calculated according to [18]). Evaluations are averaged over five samples, and the sample standard deviations are reported in parentheses.

	♘xe3	d8♖	d8♕	♗c2+	♔d5
**Q-value**	−3.62 (0.06)	−4.26 (0.69)	−4.28 (0.60)	−4.76 (0.21)	−5.12 (0.08)
**Win Prob.**	11.1% (0.4%)	8.3% (2.8%)	8.1% (2.2%)	6.1% (0.7%)	5.0% (0.2%)

**Table 2:** Original puzzle: Stockfish 14 evaluations at depth 39 for the top 5 moves (MultiPV = 5) and corresponding approximate win probabilities (calculated according to [18]). Evaluations are averaged over five samples, and the sample standard deviations are reported in parentheses.

	♘f6+	♘xe3	d8♕	d8♖	♗c2+
**Q-value**	+5.44 (1.86)	−3.98 (0.06)	−4.49 (0.51)	−4.50 (0.53)	−5.27 (0.05)
**Win Prob.**	93.8% (6.4%)	9.2% (0.3%)	7.2% (1.8%)	7.2% (2.0%)	4.6% (0.1%)

**Table 3:** Corrected puzzle: Stockfish 14 evaluations at depth 40 for the top 5 moves (MultiPV = 5) and corresponding approximate win probabilities (calculated according to [18]). Evaluations are averaged over five samples, and the sample standard deviations are reported in parentheses.

	♘f6+	♘xe3	d8♖	d8♕	♔e6
**Q-value**	*∞* (0)	−3.31 (0.07)	−4.28 (0.30)	−4.31 (0.27)	−4.92 (0.09)
**Win Prob.**	100% (0%)	13.0% (0.4%)	7.9% (1.3%)	7.8% (1.1%)	5.6% (0.3%)

**Table 4:** Corrected puzzle: LCZero evaluations for the top 5 moves, along with ♘xe3, after analysing 60 million nodes. Q-value, win probability, policy network output, fraction of visits, and estimated moves remaining are reported for each move. Evaluations are averaged over five samples, and sample standard deviations are reported in parentheses when necessary (policy network output is always constant).

	d8♖	♔c6	d8♕	♘f6+	♗c2+	♘xe3
**Q-value**	−4.67 (0.01)	−5.02 (0.01)	−5.44 (0.09)	−5.61 (0.00)	−6.46 (0.10)	−8.88 (0.00)
**Win Prob.**	5.86% (0.01%)	5.44% (0.01%)	5.02% (0.08%)	4.86% (0.00%)	4.2% (0.07%)	3.01% (0.00%)
**Policy**	3.11%	7.23%	13.33%	7.40%	9.22%	15.75%
**Visits**	0.53% (0.00%)	2.39% (0.04%)	1.27% (0.01%)	0.36% (0.00%)	1.07% (0.03%)	92.35% (0.05%)
**Moves left**	73.6 (0.1)	79.3 (0.1)	73.8 (0.1)	63.7 (0.0)	80.5 (0.6)	95.9 (0.0)

## Data Availability

The data used in this study are available in Figure 1.
